# The Heart Had More to Reveal: Left Atrial Myxoma With Concomitant Coronary Artery Disease in a Hispanic Woman

**DOI:** 10.7759/cureus.116996

**Published:** 2026-09-27

**Authors:** Yomara Huertas Gomez, Robert Rodriguez Cruz, Robert Ferrer Macias, Jose Roman-Ramos, Andres Aviles Candelario

**Affiliations:** 1 Internal Medicine, Centro Médico Episcopal San Lucas, Ponce, PRI; 2 Cardiology, Centro Médico Episcopal San Lucas, Ponce, PRI; 3 Cardiology, Hospital Episcopal San Lucas, Ponce, PRI

**Keywords:** cardiac multimodality imaging, cardiac myxoma, coronary artery disease, coronary revascularization, junctional bradycardia

## Abstract

Primary cardiac tumors are rare, with cardiac myxomas representing the most common benign primary cardiac neoplasm. Although surgical excision is definitive, comprehensive evaluation is essential to characterize the mass and identify concomitant cardiovascular disease that may alter management. We present an 86-year-old woman with hypertension, type 2 diabetes mellitus, hypothyroidism, and a 10-pack-year smoking history who was referred after transthoracic echocardiography (TTE) identified a left atrial mass concerning for myxoma versus thrombus. She reported intermittent exertional chest tightness. A prior echocardiogram in 2023 demonstrated preserved left ventricular ejection fraction (LVEF) >55% without significant structural heart disease. Current TTE showed a mildly dilated left ventricle, mild concentric hypertrophy, global hypokinesis, LVEF 40%-45%, and a left atrial mass attached to the interatrial septum. Transesophageal echocardiography further characterized the lesion as a mobile, irregular mass arising from the interatrial septum, consistent with a left atrial myxoma, without mitral inflow obstruction. Given her age, cardiovascular risk factors, exertional symptoms, and anticipated cardiac surgery, coronary angiography was performed and revealed 70%-80% stenosis of the mid-left anterior descending artery (LAD), with preserved LV systolic function.

The patient underwent resection of the left atrial mass with septal repair and concomitant coronary artery bypass grafting using the left internal mammary artery to the LAD. Histopathology confirmed cardiac myxoma. Postoperatively, temporary epicardial pacing was required. After pacing was discontinued, she developed symptomatic junctional bradycardia at <40 beats/min associated with dizziness, nausea, and malaise. Persistent symptomatic bradyarrhythmia ultimately required permanent dual-chamber pacemaker implantation. This case highlights the complexity of managing cardiac myxoma in an elderly patient with concomitant coronary artery disease. Multimodality imaging established the diagnosis and defined tumor anatomy, while preoperative coronary evaluation identified significant LAD disease and enabled simultaneous tumor resection and revascularization. Close postoperative rhythm surveillance is also essential, as clinically significant bradyarrhythmias may require permanent pacing. A multidisciplinary approach is critical to individualizing management of these rare cardiac tumors.

## Introduction

Primary cardiac tumors are exceedingly rare, with a reported incidence ranging from 0.0017% to 0.19%. Approximately 75% are benign, with cardiac myxomas representing the most common primary cardiac neoplasm [1]. More than 75% of myxomas arise in the left atrium, characteristically originating from the interatrial septum near the fossa ovalis [2]. Their clinical presentation is highly variable, ranging from incidental detection to the classic triad of intracardiac obstruction, systemic embolization, and constitutional symptoms [2,3]. Although surgical excision remains the definitive treatment, comprehensive preoperative evaluation is essential to characterize tumor anatomy, assess hemodynamic consequences, and identify concomitant cardiovascular disease that may influence the operative strategy. In patients undergoing cardiac surgery, particularly older individuals and those with cardiovascular risk factors or symptoms suggestive of ischemia, assessment for concomitant coronary artery disease forms an important component of preoperative planning [3,4].

We present a case of left atrial myxoma in an elderly patient managed at a tertiary care hospital in Puerto Rico, in whom multimodality cardiovascular evaluation revealed significant concomitant coronary artery disease. This finding ultimately led to combined tumor resection and coronary revascularization, followed by postoperative symptomatic bradyarrhythmia requiring permanent pacemaker implantation.

## Case presentation

An 86-year-old woman with a medical history of essential hypertension, type 2 diabetes mellitus, and hypothyroidism was referred by her outpatient cardiologist for further evaluation of a newly identified left atrial mass on transthoracic echocardiography (TTE), initially concerning for an atrial myxoma versus intracardiac thrombus. She reported intermittent exertional chest tightness and had a 10-pack-year smoking history. A prior transthoracic echocardiogram performed in 2023 demonstrated preserved left ventricular systolic function with a left ventricular ejection fraction (LVEF) >55%, normal diastolic function, trace mitral and tricuspid regurgitation, normal estimated right ventricular systolic pressure, and a normal inferior vena cava.

During the initial evaluation, transthoracic echocardiography (TTE) demonstrated a mildly dilated left ventricle with mild concentric left ventricular hypertrophy, mildly reduced left ventricular systolic function with an estimated left ventricular ejection fraction (LVEF) of 40%-45%, and mild global hypokinesis (Figure 1). Grade I diastolic dysfunction with normal estimated left atrial pressure was also present. A left atrial mass attached to the interatrial septum was identified, raising concern for an atrial myxoma versus thrombus. Moderate right atrial enlargement was additionally noted.

**Figure 1:**
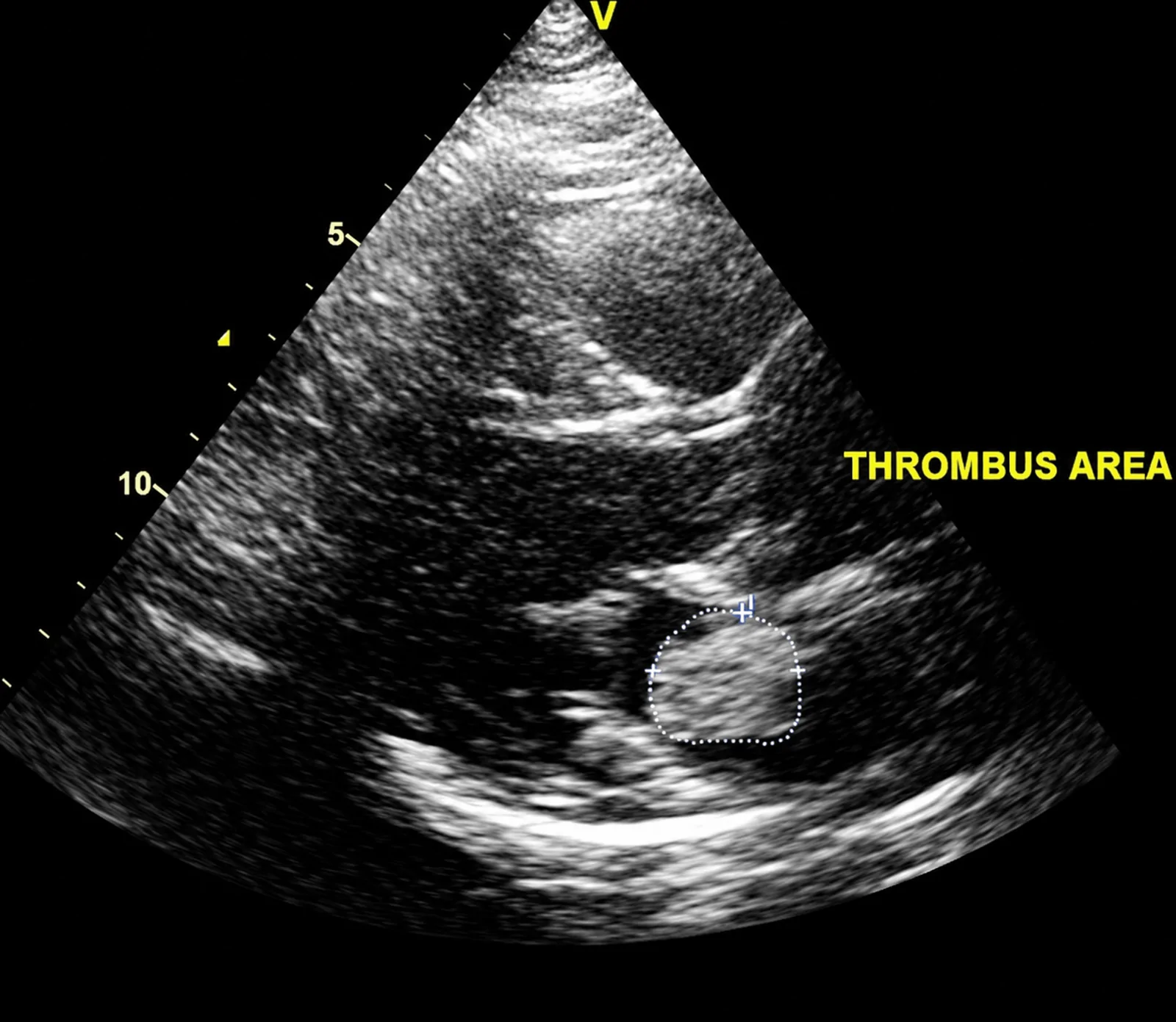
Transthoracic echocardiography, parasternal long-axis view, demonstrating the area of atrial mass (dotted outline).

Given the uncertain nature of the intracardiac mass, transesophageal echocardiography (TEE) was subsequently performed for further characterization. TEE demonstrated a 4 × 6 cm mobile left atrial mass attached to the interatrial septum with irregular borders and morphologic features consistent with a left atrial myxoma. The mass did not prolapse across the mitral valve and produced no functional mitral inflow obstruction (Figure 2). No left atrial appendage thrombus was identified. The interatrial septum was intact by color Doppler and agitated saline contrast study. TEE demonstrated preserved left ventricular systolic function with an LVEF >55%, mild concentric left ventricular hypertrophy, normal right ventricular size and systolic function, and mild mitral and tricuspid regurgitation. The aortic valve was sclerotic and trileaflet without significant stenosis or regurgitation.

**Figure 2:**
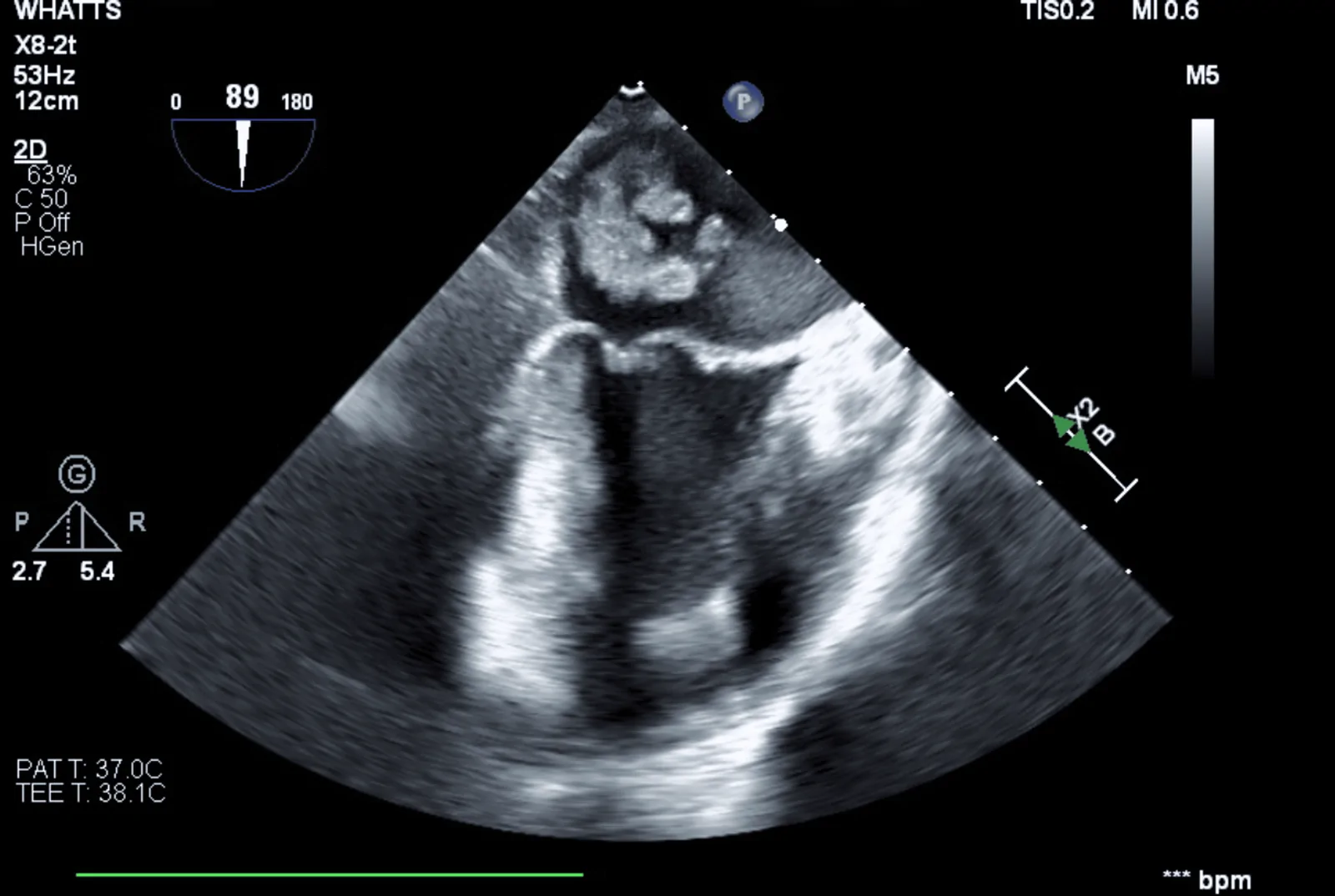
Transesophageal echocardiography (TEE) in the mid-esophageal bicaval view at approximately 90° demonstrating a large, heterogeneous, mobile left atrial mass attached to the interatrial septum, consistent with a left atrial myxoma.

Cardiothoracic surgery was consulted for consideration of surgical resection of the presumed left atrial myxoma. Given the patient's advanced age, cardiovascular risk factors, exertional chest symptoms, and anticipated cardiac surgery, a comprehensive preoperative cardiovascular evaluation was undertaken.

Thereafter, left heart catheterization with coronary angiography demonstrated significant single-vessel coronary artery disease, characterized by 70%-80% stenosis of the mid-left anterior descending coronary artery. Left ventricular systolic function was preserved, with an estimated LVEF >60% (Figures 3, 4).

**Figure 3:**
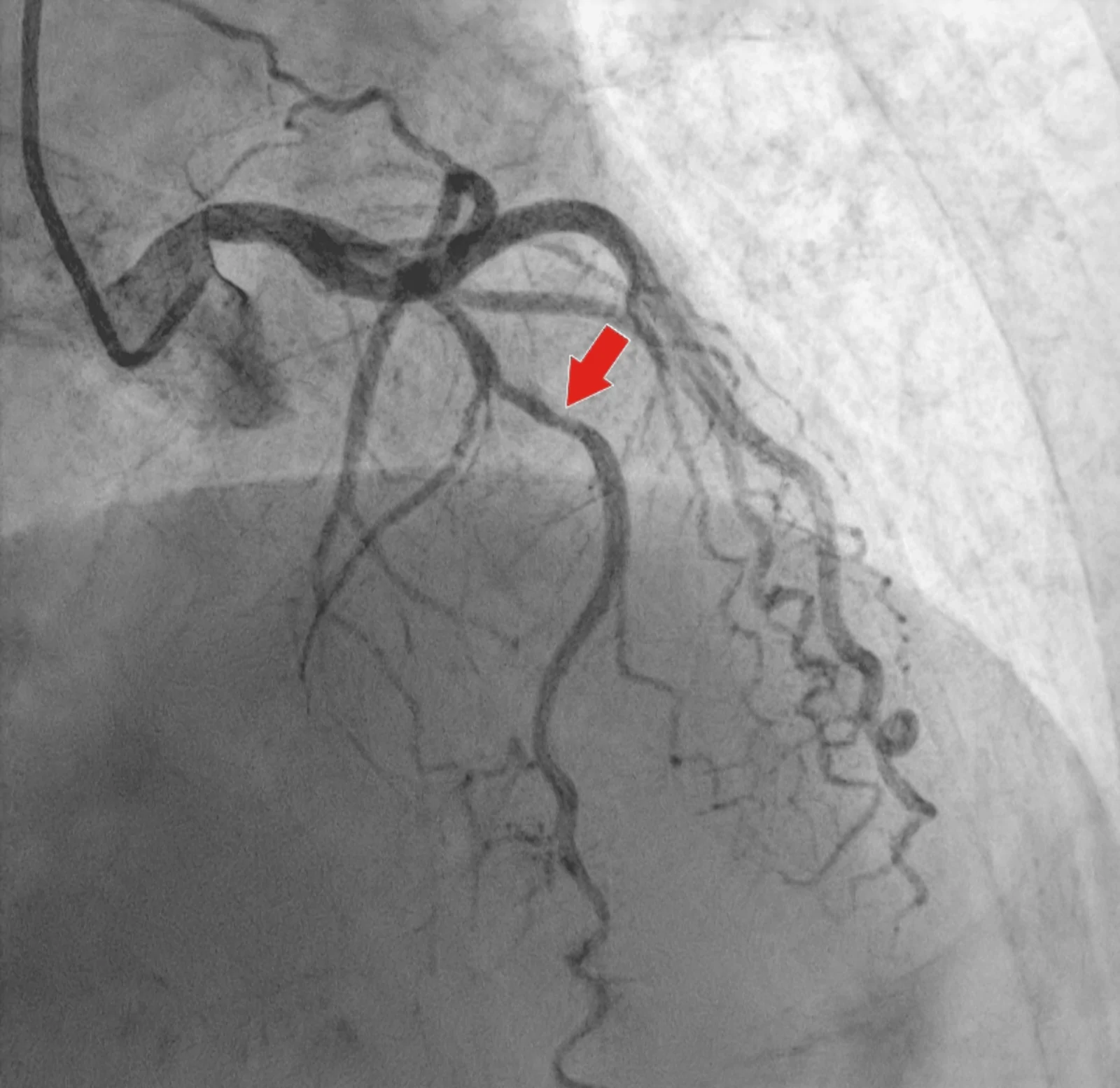
Left coronary angiography in a left anterior oblique (LAO) caudal projection demonstrating severe stenosis of the mid-left anterior descending (LAD) coronary artery (red arrow).

**Figure 4:**
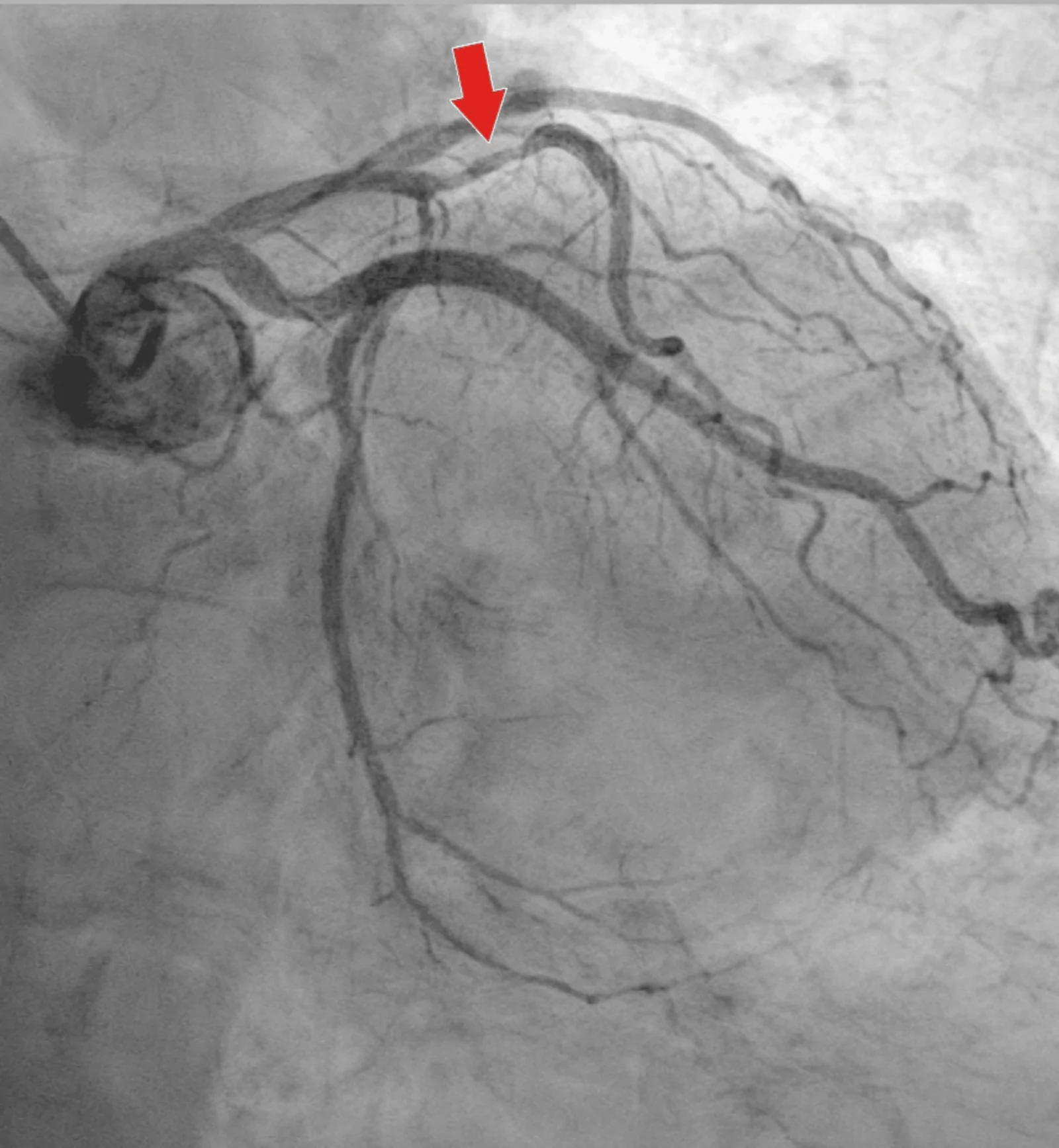
Coronary angiography in a right anterior oblique (RAO) caudal projection demonstrating significant stenosis of the mid-left anterior descending (LAD) coronary artery (red arrow).

There was discordance in the assessment of left ventricular systolic function across imaging modalities. The initial TTE estimated an LVEF of 40%-45% with mild global hypokinesis, whereas subsequent TEE demonstrated an LVEF >55%, and left ventriculography during cardiac catheterization demonstrated preserved systolic function with an estimated LVEF >60%. This discrepancy may reflect differences in imaging modality, loading conditions, and the inherent variability of LVEF estimation. Because serial quantitative measurements using the same imaging modality were not available, the precise reason for this discrepancy could not be definitively established.

Histological study of the resected left atrial mass showed cardiac myxoma with positive staining for calretinin and vimentin. Histological study of the resected interatrial septum revealed myocyte hypertrophy and mild chronic inflammation in an elderly patient with concomitant significant obstructive coronary artery disease, requiring multidisciplinary assessment to determine the optimal surgical strategy. 

The patient underwent surgical resection of the left atrial mass with repair of the resulting atrial septal defect and concomitant single-vessel coronary artery bypass grafting (CABG) using the left internal mammary artery (LIMA) to the LAD. Histopathologic examination confirmed cardiac myxoma. Postoperatively, temporary epicardial pacing in dual-chamber paced, dual-chamber sensed, and dual-response-to-sensing (DDD) mode was required. After temporary pacing was discontinued, an underlying sinus rhythm of approximately 60 beats/min was initially observed. The patient subsequently developed junctional bradycardia at <40 beats/min associated with dizziness, nausea, and malaise. Because the symptomatic bradyarrhythmia persisted, a permanent dual-chamber pacemaker was ultimately implanted.

## Discussion

Cardiac myxomas are rare benign primary cardiac tumors that may nevertheless result in serious cardiovascular complications because of their intracardiac location, mobility, and embolic potential. Once identified, complete surgical excision remains the treatment of choice, particularly because delayed intervention may increase the risk of systemic embolization or hemodynamic compromise [1,4]. A 2024 meta-analysis of 112 studies involving 8,150 patients demonstrated that 85.3% of cardiac myxomas were located in the left atrium, with generally favorable surgical outcomes, an early mortality of approximately 1.27%, and a low long-term recurrence rate [4]. In our patient, the tumor arose from the interatrial septum and was mobile but did not prolapse across the mitral valve or produce functional mitral inflow obstruction. Complete surgical resection was performed, and histopathologic examination confirmed the diagnosis of cardiac myxoma.

Although epidemiologic data regarding cardiac tumors in Puerto Rico remain limited, the available regional literature demonstrates patterns similar to those reported internationally. A retrospective study from the Cardiovascular Center of Puerto Rico and the Caribbean identified 77 patients with a diagnosis of benign cardiac neoplasm between 1992 and 2008, of whom 43 had pathologic confirmation. Myxomas accounted for 83.7% of confirmed tumors, and 81% of masses were located in the left atrium [5]. The mean age at diagnosis was 51.5 years, and the most common clinical presentations were heart failure (35%), chest pain (18%), and neurologic manifestations (14%). Surgical complications occurred in 30% of patients, with an overall mortality of 7% [5]. These regional findings provide relevant context for our 86-year-old woman patient, who was evaluated and treated at a tertiary care hospital in Puerto Rico and presented with exertional chest discomfort and a left atrial mass. Her advanced age and subsequent complex cardiovascular course distinguish her presentation from the average patient described in the local cohort.

An important feature of this case was the identification of concomitant obstructive coronary artery disease during the preoperative evaluation. Although the left atrial mass was the primary indication for cardiac surgery, the patient's advanced age, hypertension, diabetes mellitus, smoking history, and exertional chest discomfort warranted further ischemic evaluation. Coronary angiography demonstrated 70%-80% stenosis of the mid-left anterior descending (LAD) coronary artery. Preoperative coronary angiography in appropriately selected patients with cardiac myxoma may identify clinically important concomitant coronary artery disease and provide information that directly influences operative planning [1,3]. In our patient, identification of significant LAD disease allowed both conditions to be addressed during the same surgical intervention.

The patient underwent complete resection of the left atrial myxoma with repair of the resulting atrial septal defect and concomitant single-vessel coronary artery bypass grafting (CABG) using the left internal mammary artery (LIMA) to the LAD. This approach provided definitive treatment of the cardiac tumor and coronary revascularization during a single operation. The case emphasizes that evaluation of a cardiac myxoma should extend beyond characterization of the intracardiac mass. In elderly patients, particularly those with ischemic symptoms or cardiovascular risk factors, evaluation for concomitant coronary artery disease may substantially alter the operative strategy.

Another clinically relevant aspect of this case was the development of symptomatic bradyarrhythmia following surgery. Arrhythmias and conduction disturbances have been reported after cardiac myxoma resection and may include atrial fibrillation, atrial flutter, junctional rhythms, sinus node dysfunction, and atrioventricular block [6,7]. A recent 20-year surgical series reported new arrhythmias or conduction disturbances in 21.1% of patients during the early postoperative period. Most were transient; however, a small proportion required long-term therapy or permanent pacemaker implantation [6]. Earlier observations specifically involving left atrial myxoma resection have also documented junctional rhythms, sinus arrest, and complete heart block, with some patients ultimately requiring permanent pacing [7].

The mechanisms responsible for postoperative conduction abnormalities following myxoma resection are likely multifactorial and remain incompletely understood. Surgical manipulation of the atrial tissue and interatrial septum may contribute through local edema, inflammation, or direct injury involving atrial conduction pathways. Cardiopulmonary bypass, perioperative myocardial ischemia, autonomic changes, and pre-existing age-related degeneration of the conduction system may also contribute [7,8]. In this patient, advanced age and surgical manipulation of the interatrial septum during myxoma resection may have contributed to the postoperative conduction disturbance; however, this single case does not establish a definitive mechanism for the junctional bradycardia.

Temporary epicardial pacing in DDD mode was initially required postoperatively. After discontinuation of pacing, an underlying sinus rhythm of approximately 60 beats/min was initially observed; however, the patient subsequently developed junctional bradycardia at <40 beats/min, accompanied by nausea, dizziness, and malaise. The temporal relationship between the bradyarrhythmia and her symptoms supported its clinical significance. However, many postoperative conduction abnormalities are transient; persistent symptomatic bradycardia warrants careful assessment. In this patient, persistent symptomatic junctional bradycardia ultimately required permanent dual-chamber pacemaker implantation.

This case from Puerto Rico highlights several important considerations in the management of cardiac myxoma. Multimodality cardiovascular imaging is essential for characterizing an intracardiac mass, defining its attachment and mobility, and assessing its hemodynamic consequences. Equally important, preoperative cardiovascular evaluation should be individualized, particularly in elderly patients with cardiovascular risk factors or ischemic symptoms, as concomitant coronary artery disease may significantly alter surgical planning. In our patient, identification of significant LAD disease permitted simultaneous tumor resection and coronary revascularization. Finally, careful postoperative rhythm surveillance remains essential because conduction disturbances, although often transient, may become symptomatic and require permanent pacing. In the context of the limited published experience with cardiac myxomas in Puerto Rico, this case adds valuable regional clinical experience and illustrates the importance of multidisciplinary collaboration among cardiovascular imaging, interventional cardiology, cardiothoracic surgery, pathology, and electrophysiology in the management of complex cardiac myxoma.

## Conclusions

Cardiac myxomas are rare benign tumors with potentially serious hemodynamic, embolic, and perioperative consequences. This case illustrates the application of a comprehensive and multidisciplinary approach in an elderly patient with a left atrial myxoma and concomitant coronary artery disease. In this patient, preoperative coronary evaluation performed in the setting of advanced age, cardiovascular risk factors, exertional symptoms, and anticipated cardiac surgery identified significant coronary artery disease and enabled simultaneous tumor resection and coronary revascularization. The postoperative development of symptomatic junctional bradycardia requiring permanent pacemaker implantation further demonstrates the relevance of postoperative rhythm surveillance in this clinical setting.

Published data on cardiac myxomas in Puerto Rico remain limited. This case contributes to the regional literature by describing the complex management of an elderly Puerto Rican patient with a left atrial myxoma, concomitant obstructive coronary artery disease, and postoperative symptomatic bradyarrhythmia requiring permanent pacemaker implantation. These findings are consistent with established principles of preoperative cardiovascular evaluation, multidisciplinary surgical planning, and postoperative monitoring rather than representing new clinical recommendations.
